# Asymmetrical nasal high flow ventilation improves clearance of CO_2_ from the anatomical dead space and increases positive airway pressure

**DOI:** 10.1152/japplphysiol.00692.2022

**Published:** 2023-01-12

**Authors:** Stanislav Tatkov, Monique Rees, Anton Gulley, Lotte G. T. van den Heuij, Georg Nilius

**Affiliations:** ^1^Fisher & Paykel Healthcare Ltd., Auckland, New Zealand; ^2^Evang. Kliniken Essen-Mitte GmbH, Essen, Germany; ^3^Universität Witten/Herdecke, Witten, Germany

**Keywords:** asymmetrical, dead space, nasal cannula, nasal high flow, respiratory support

## Abstract

Positive airway pressure that dynamically changes with breathing, and clearance of anatomical dead space are the key mechanisms of noninvasive respiratory support with nasal high flow (NHF). Pressure mainly depends on flow rate and nare occlusion. The hypothesis is that an increase in asymmetrical occlusion of the nares leads to an improvement in dead-space clearance resulting in a reduction in re-breathing. Clearance was investigated with volumetric capnography in an adult upper-airway model, which was ventilated by a lung simulator with entrained carbon dioxide (CO_2_) at respiratory rates (RR) of 15–45 min^−1^ and at 18 min^−1^ with chronic obstructive pulmonary disease (COPD) breathing patterns. Clearance was assessed at NHF of 20–60 L/min with a symmetrical interface (SI) and an asymmetrical interface (AI). CO_2_ kinetics visualized by infrared spectroscopy and mathematical modeling were used to study the mechanisms of clearance. At a higher RR (35 min^−1^) and NHF of 60 L/min, clearance in the upper airway was significantly higher with the AI when compared with the SI (29.64 ± 9.96%, *P* < 0.001), as opposed to at a lower RR (15 min^−1^) (1.40 ± 6.25%, *P* > 0.05), (means ± SD). With COPD breathing, clearance by NHF was reduced but significantly improved with the AI by 45.93% relative to the SI at NHF 20 L/min (*P* < 0.0001). The maximum pressure achieved with the AI was 6.6 cmH_2_O and NHF was 60 L/min at the end of expiration. Pressure differences between nasal cavities led to the reverse flow observed in the optical model. Asymmetrical NHF increases dead-space clearance by reverse flow through the choanae and accelerates purging of expired gas via the less occluded nare.

**NEW & NOTEWORTHY** The asymmetrical interface generated reverse flow in the nasal cavities and across the choana, which led to unidirectional purging of expired gas from the upper airways. This accelerated the clearance of anatomical dead space and reduced re-breathing while increased resistance to flow resulted in higher positive end-expiratory pressure (PEEP). These findings are relevant to patients with elevated respiratory rates or with expiratory flow limitations where dead-space clearance by NHF can be substantially reduced.

## Graphical abstract

**Figure:**
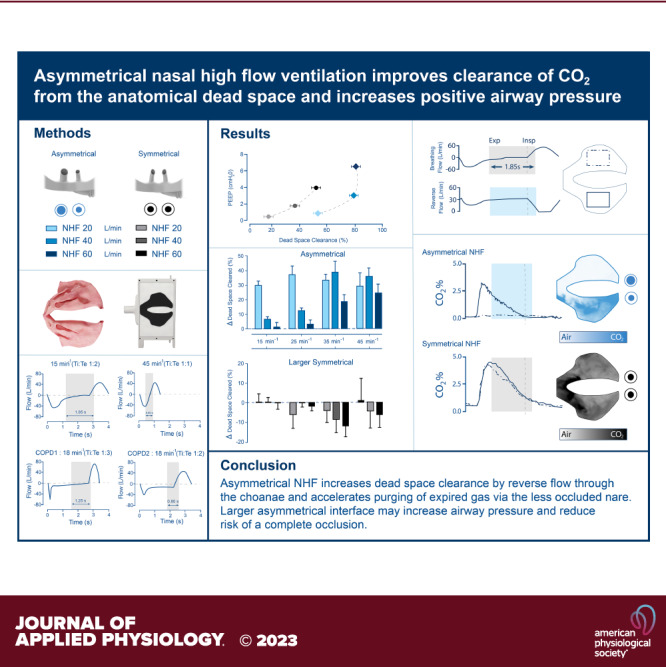

## INTRODUCTION

Nasal high flow (NHF) therapy is an established form of noninvasive respiratory support used in acute and chronic care by delivering heated and humidified air with or without supplemental oxygen via a nasal cannula interface (1, 2). NHF has become the standard of care in patients with acute respiratory failure with a typical flow range between 20 and 60 L/min and up to 80 L/min in adults and has been used extensively in patients with COVID-19 (3–5).

Positive airway pressure that dynamically changes with respiratory flow and reduced re-breathing of expired gas from the upper airways are believed to be the key mechanisms of NHF (6, 7). This distinguishes it from other forms of pressure-controlled therapies, such as continuous positive airway pressure (CPAP) or noninvasive ventilation (NIV). Note that for NIV to be effective in acute respiratory distress syndrome (ARDS), a minimum positive end-expiratory pressure (PEEP) of 5 cmH_2_O is needed (8). PEEP during NHF therapy mainly depends on the flow rate and resistance to flow, which is determined by nare occlusion (7). A larger cannula with a greater cross-sectional area can increase PEEP during NHF but may risk fully occluding the nares (9, 10). With the mouth closed this could result in an uncontrollable rise in airway pressure as well as the inability to breathe through the nose. Barotrauma in patients during NHF has been reported in past literature (11, 12).

A reduction in re-breathing based on dilution and purging of expired gas from the upper airways by NHF improves gas exchange (13, 14). This is achieved by lowering dead-space ventilation and is linked to various physiological outcomes, including a reduction in the work of breathing (2, 7). Dead-space clearance is flow- and time dependent and is reduced at higher respiratory rates (RRs), mainly due to shortened expiratory time, which is commonly observed in acute respiratory failure. A reduction of RR leads to increased clearance of dead space, improving gas exchange and further lowering breathing frequency. This was proposed as a rationale for higher NHF settings in patients with tachypnea being beneficial (7). In obstructive lung diseases like chronic obstructive pulmonary disease (COPD), where expiratory flow limitation may affect breathing pattern, the effect on dead-space clearance efficiency has not yet been investigated. The effect of the nasal prong size on dead-space clearance is not fully understood. Smaller-sized prongs increase gas velocity but may reduce pressure due to increased leak, and there is conflicting data about the effect this has on dead-space clearance (7, 15, 16). Single-pronged cannulae have been investigated and were found to improve gas exchange in patients with hypercapnic COPD (17).

The novel asymmetrical nasal cannula interface (AI), when compared with the standard symmetrical nasal cannula interface (SI), may increase PEEP while mitigating the risk of full occlusion due to different prong diameters. The hypothesis is that an increase in asymmetrical occlusion of the nares leads to an improvement in dead-space clearance that resulted in a reduction in re-breathing.

## MATERIALS AND METHODS

The upper airway model was based on averaged adult European upper-airway geometry with the smallest cross-sectional area in the vestibulum nasi at 78.5 mm^2^ per nare (total 157 mm^2^) (18, 19). In a second model, the nares size was upscaled to 170% to test the effect of a larger leak area. The models were fabricated out of Somos GP Plus 14122 resin (DSM, P.R. China) using a Hontai Stereolithography three-dimensional (3-D) printer (Supplemental Material). In bench top experiments, the commercially available symmetrical cannula interface (SI) (Optiflow+, Fisher & Paykel Healthcare Ltd., New Zealand) and asymmetrical cannula interface (AI) (Optiflow Duet, Fisher & Paykel Healthcare Ltd., New Zealand) were used in an anatomically correct three-dimensionally printed upper airway model connected to a lung simulator (ASL 5000, IngMar Medical) to measure airway pressure and re-breathing using volumetric capnography. The ASL 5000 was used to generate a consistent breathing pattern in the SmartPump mode to ensure the flow patterns were controlled and consistent despite changes to resistance. Carbon dioxide (CO_2_) was entrained to simulate expiratory gas concentrations using a low flow of 100% CO_2_ titrated into the piston of the ASL through a perforated flexible tube. NHF at 20, 40, and 60 L/min was generated using a commercially available integrated flow generator and humidifier (Airvo 3, Fisher & Paykel Healthcare Ltd., New Zealand).

**Figure 1.**
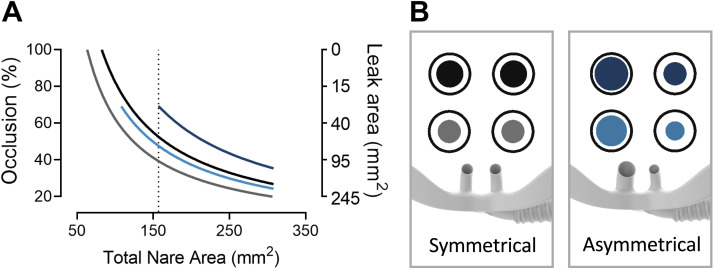
*A*: the relationship between total nare area, percent occlusion, and total leak area by the large (black OPT 946) and medium (gray OPT944) symmetrical interface (SI) and asymmetrical large (dark blue, OPT966) and medium (light blue OPT964) interfaces (AI) used in the study. Reduction of the nare area may lead to a complete occlusion of both nares with the SI, which is less likely with the different-sized prongs of the AI. The dotted line represents the averaged adult nare area used in the model. *B*: a schematic view of the prong’s cross-sectional area (internal circles) and the nares (external circles) with the leak area as a gap between the internal and external circles; these are depicted in proportion.

The AI has asymmetrical prongs; the right prong diameter has been reduced and the left prong diameter has been increased compared with the SI. This increases the total cross-sectional area of both prongs by 30% to 40% (Fig. 1). The large SI and the medium AI are compared as these have a similar occlusion area and block ∼50% of the model’s nares (Fig. 1). Gray and blue inner circles represent prongs in SI and AI, respectively. The larger AI, which almost completely occludes one nare in the model, was used to investigate the highest achievable resistance and maximum airway pressure values.

An optically transparent model was used to visualize the kinetics of CO_2_ clearance by infrared spectroscopy (7, 13). In this model, the nasal cavities were positioned in the horizontal plane with symmetrical reflection and sandwiched between two sapphire optical windows (Supplemental Video S1). Breathing was simulated using a lung simulator as before. Both models had pressure ports located in the nasal cavities (20 mm from the external nares) to measure the differential pressure between cavities.

To study the effect of increased RRs on the clearance of dead space, a RR of 15, 25 min^−1^ (Ti:Te 1:2) and a RR of 35, 45 min^−1^ (Ti:Te 1:1) were used. To study the effect of breathing patterns on the clearance of dead space, the following simulated breathing patterns were used: patients with stable COPD with expiratory flow limitation at a RR of 18 min^−1^, COPD 1 with a prolonged expiration (Ti:Te 1:3) and COPD 2 (Ti:Te 1:2) with intrinsic PEEP, characterized by a higher expiratory flow just before expiration ended. A normal breathing pattern (Ti:Te 1:2) was taken as the control. The time to clear 100 mL at the end of expiration and the beginning of inspiration was used as the time available for dead-space clearance in the upper airways, as described previously in detail (7). The time to expire the last and inspire the first 100 mL was when majority of the dead-space clearance occurred in adult upper airways. For every breathing pattern tested, the lung simulator produced 10 breaths of which the first and last two breaths were excluded from the analysis to avoid any transitionary artifacts; the six most stable middle breaths were used.

Breathing by an operator via the optical model was used to replicate the effect of a variable respiratory pattern on the dead-space clearance with different interfaces and NHF rates. Re-breathing at the level of the trachea (Supplemental Material) was quantified using volumetric capnography (OG-3800K and TG-980P, Nihon Kohden, Japan and Fleisch Pneumotachograph, Size 2, range ± 2.5 L/s, Switzerland) and the clearance kinetics in the nasal cavities was investigated using infrared spectroscopy (FLIR X6998SC, Teledyne FLIR LLC) with an integrated cooled CO_2_ filter and recorded at 500 frames/s. A panel coated with Vantablack (Surrey NanoSystems, UK) and heated to 150°C served as a source of homogeneous midinfrared radiation. Both methods have been described in detail previously (7).

Mathematical modeling was based on the Wheatstone bridge circuit and known empirical fluid dynamic relationships for bulk flow in channels (20). The Darcy–Weisbach equation was used to model the turbulent flow in the cannula prongs $\Delta P=RQ^{2}\;$and the Hagen–Poiseuille equation was used for flow in the airways ΔP=RQ (P—pressure, R—resistance, and Q—flow). The computations were performed using MATLAB software (MathWorks) to predict flow and pressure differences in the nasal cavities during breathing and are described in more detail in the Supplemental Material.

### Data Analyses

The analog signals of pressure, flow, and CO_2_ concentration were digitized using a 16-bit ADC (ADI PowerLab 16/30; ADInstruments, New Zealand) and recorded in LabChart 8 at 1,000 Hz. Re-breathed dead space was calculated by integrating the inspired flow with CO_2_ and dividing by the end-tidal CO_2_ as measured by the pneumotachometer and capnograph (7). Raw data were exported to MATLAB for analysis.

Six breaths were analyzed per breathing pattern to find the mean PEEP and re-breathed volume. All re-breathed volumes were normalized to the “no NHF” condition for each RR to give a percentage of improved dead-space clearance from baseline.

Infrared spectroscopy was recorded using Research IR and analyzed in Research Studio (Teledyne FLIR LLC). Two regions of interest (ROIs) were defined by bounding boxes within the upper and lower nare cavities of the optical model. The mean saturation of each ROI was calculated per frame and time was aligned to match the analog data from LabChart. A linear relationship was assumed between infrared absorption and CO_2_ concentration as the optical length was constant across all experiments. A calibration factor was calculated using the maximum and minimum baseline CO_2_ concentration conditions. This was used to convert all subsequent ROI values so that changes in CO_2_ clearance could be visualized per nasal cavity.

The statistical analysis was performed by Prism V.9 (GraphPad Software) using two-way ANOVA, Mann–Whitney *U*, or paired *T* test, where applicable, and the threshold for statistical significance was set at *P* < 0.05. Data are presented as means ± SD.

## RESULTS

### Effect of Increased Asymmetrical versus Symmetrical Occlusion on Clearance of Dead Space

**Figure 2.**
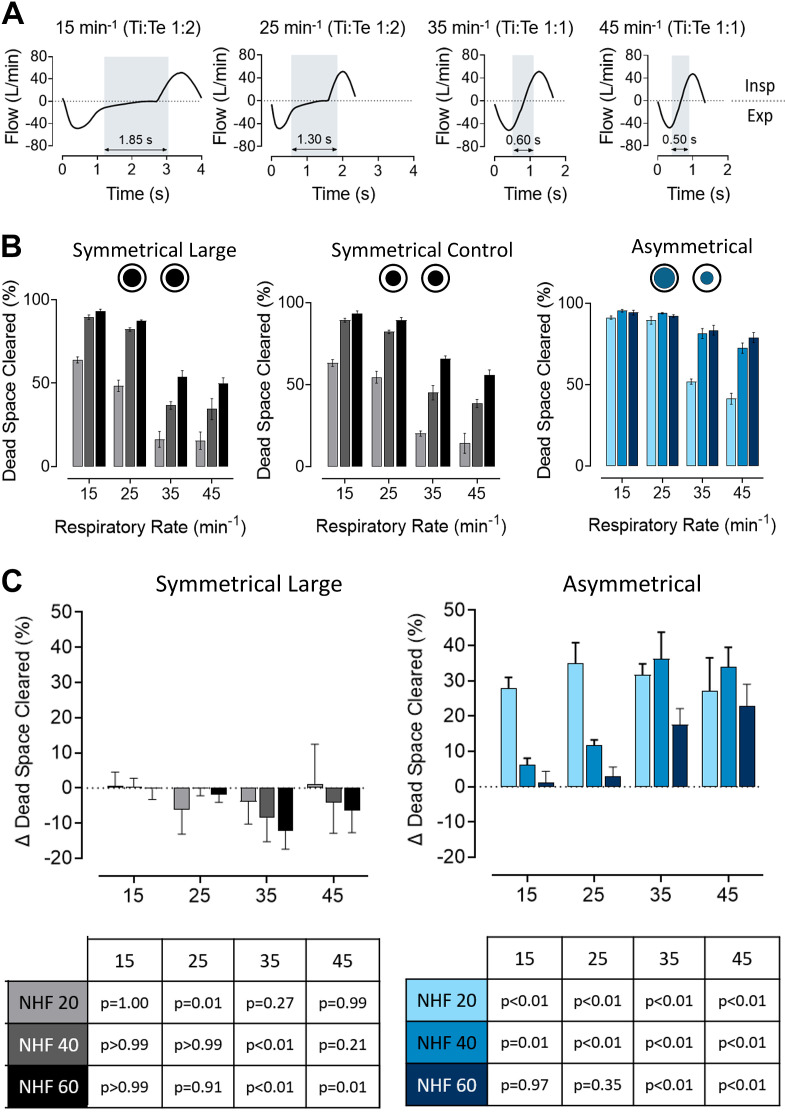
Effect of an increased occlusion by symmetrical cannula interface (SI) and asymmetrical cannula interface (AI) on dead-space clearance in the anatomically correct three-dimensional upper airway model during nasal high flow (NHF) rates of 20, 40, and 60 L/min and an increasing respiratory rate (RR). *A*: breathing patterns used in the lung simulator at RRs of 15, 25, 35, and 45 min^−1^. Gray vertical bars demonstrate time of the last and first 100 mL during expiration and inspiration. This corresponds to the time when the majority of clearance occurs. An increase in RR leads to reduced clearance time. *B*: clearance achieved by increased total nare occlusion by an SI (*left*) and AI (*right*). A standard medium-sized SI was used as control, as shown in the central Fig. *C*: this illustrates the difference in clearance due to increased nare occlusion with SI (*left*) and AI (*right*) relative to the SI control. Asymmetrical occlusion increases dead-space clearance. Symmetrical occlusion leads to reduced clearance at higher RRs where the difference in clearance between the two interfaces is the greatest. At an RR of 15 min^−1^ and NHF of 60 L/min, the difference is not significant (see *P* value table in graph).

A comparison of symmetrical and asymmetrical interfaces showed increased total nare occlusion. Examples of the simulated breathing patterns used in the subsequent graphs are depicted in Fig. 2*A*, in which the last 100 mL of expiration and first 100 mL of inspiration are shaded in gray, highlighting the reduction in time for dead-space clearance as the RR increases from 15 to 45 min^−1^. In Fig. 2*B*, the central graph shows normalized data for dead-space clearance at increasing RRs using a standard medium SI. There is a reduction in dead-space clearance with increasing RR. The outside graphs show the dead-space clearance with a SI (*left*) and AI (*right*) with an equivalent increase in the combined cross-sectional area. The relative changes in dead-space clearance, when compared with the standard SI control, for the SI and AI with greater occlusion are shown in Fig. 2*C*. Note that increased symmetrical nare occlusion does not improve dead-space clearance, and significantly worsens it at higher RRs (*P* < 0.05). However, a similar increase in nare occlusion with the AI significantly improves dead-space clearance (*P* < 0.05). The dead space cleared at a RR of 15 min^−1^ and NHF 60 L/min was not significantly different between the SI and the AI [−1.40 ± 6.25% (*P* > 0.05)]. Nevertheless, at an RR of 35 min^−1^ the SI clearance decreased compared with the control, and the AI clearance increased, resulting in a significant difference of 29.64 ± 9.96% (*P* < 0.05).

**Figure 3.**
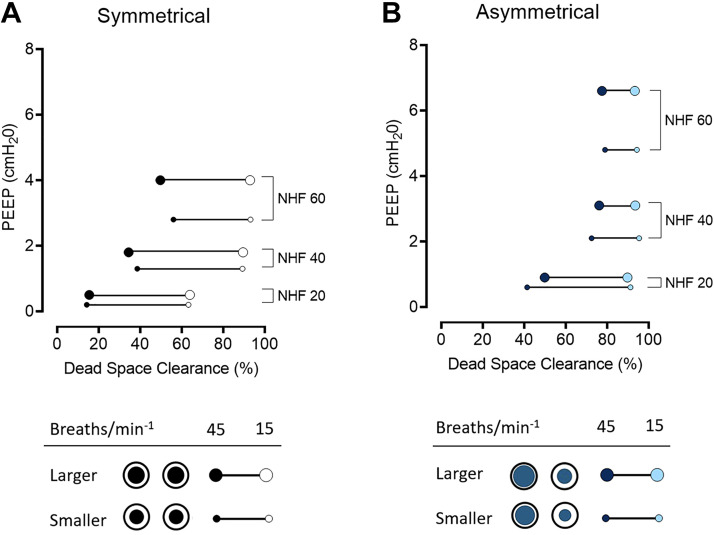
The relationship between the dead-space clearance and positive end-expiratory pressure (PEEP) measured in the upper airway model during nasal high flow (NHF) rates of 20, 40, and 60 L/min with the symmetrical cannula interface (SI; *A*) and asymmetrical cannula interface (AI; *B*) during simulated breathing at an respiratory rate (RR) of 45 min^−1^ (Ti:Te 1:1) and 15 min^−1^ (Ti:Te 1:2) for different levels of occlusion (larger and smaller cannulae). The SI at an RR of 45 min^−1^ demonstrates a more linear relationship between the clearance and PEEP when compared with the AI (*R*^2^ = 0.79 vs. *R*^2^ = 0.65 respectively).

The relationship between dead-space clearance at a RR of 15 and 45 min^−1^ and PEEP is illustrated in Fig. 3. The effect of nare occlusion with a larger and smaller cannula is demonstrated in Fig. 3*A* for the SI and in Fig. 3*B* for the AI. At a RR of 45 min^−1^, the relationship between clearance and PEEP is more linear for the SI than for the AI (*R*^2^ = 0.79 vs. *R*^2^ = 0.65, respectively) as the AI approached maximal clearance at 40 L/min and did not further increase with higher flow rates. The highest PEEP (6.6 cmH_2_O) was achieved with the larger AI at NHF of 60 L/min. Furthermore, the average difference in the amount of dead space cleared between a RR of 15 and 45 min^−1^ is significantly lower with the AI (26.8 ± 13.3%) when compared with the SI (47.3 ± 5.7%, *P* < 0.02). Results from experiments with an increased nare cross-sectional area (170%) are presented in the Supplemental Fig. S1.

**Figure 4.**
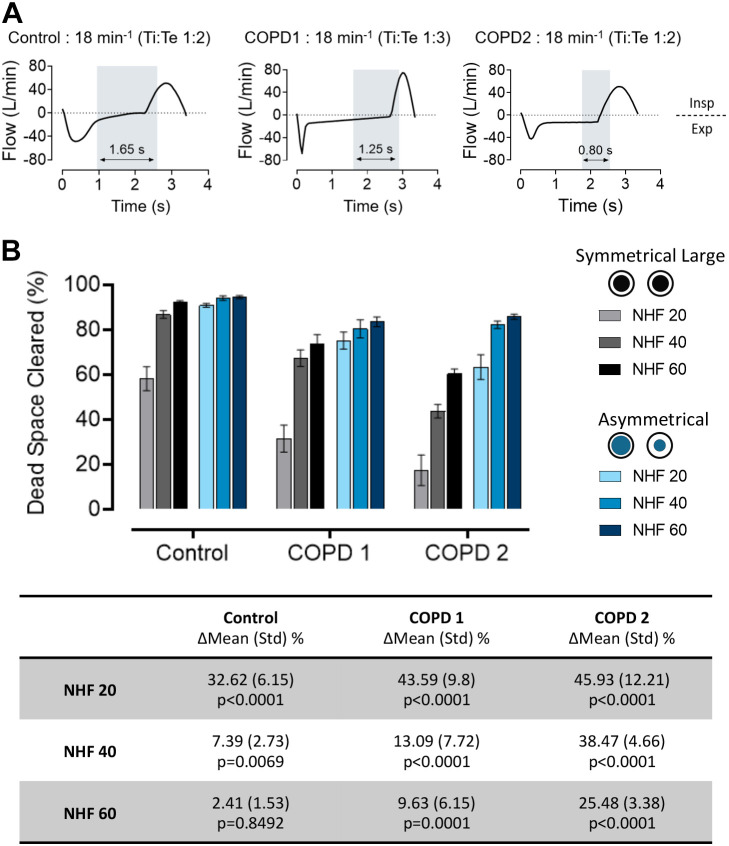
Effect of simulated breathing patterns at an respiratory rate (RR) of 18 min^−1^ on the dead-space clearance with the symmetrical cannula interface (SI) and asymmetrical cannula interface (AI) with nasal high flow (NHF) rates of 20, 40, and 60 L/min. *A*: breathing patterns with (chronic obstructive pulmonary disease, COPD 1 and 2) and without (control) expiratory flow limitations used in the experiment. COPD 2 also generates “intrinsic positive end-expiratory pressure (PEEP)” and has higher expiratory flow toward the end of expiration, but with the same Ti:Te ratio as in the control. Gray vertical bars demonstrate the time of the last and first 100 mL during expiration and inspiration where most of dead-space clearance occurs. *B*: in COPD 1 and 2 the clearance achieved with the SI is reduced; the clearance by AI was significantly less affected by the reduction in clearance time. The dead-space clearance with the SI at NHF of 60 L/min and the AI at 20 L/min were not significantly different. The table shows the differences in the average percentage of clearance between the AI and SI at the different flow rates used within each group (means ± SD). The breathing patterns for both COPD groups showed a reduced clearance time regardless of the Ti:Te ratio, resulting in a reduction in dead-space clearance, which was significantly improved by NHF with the AI.

Examples of the simulated breathing patterns at a RR of 18 min^−1^ are shown in Fig. 4*A*. The last 100 mL of expiration and first 100 mL of inspiration are shaded in gray, highlighting the reduction in time for dead-space clearance between normal and COPD-like breathing patterns. In Fig. 4*B*, in COPD 1 and 2 with expiratory flow limitation, the decreased time for clearance resulted in a significant reduction in dead space cleared with the SI. The AI performed significantly better at NHF of 20 and 40 L/min (*P* < 0.006) in all groups, and at NHF 60 L/min in COPD 1 (*P* < 0.005) and COPD 2 (*P* < 0.001). Furthermore, there is no significant difference in dead-space clearance between the SI at NHF 60 L/min and AI at NHF 20 L/min.

### Kinetics of Dead-Space Clearance during Asymmetrical Occlusion

**Figure 5.**
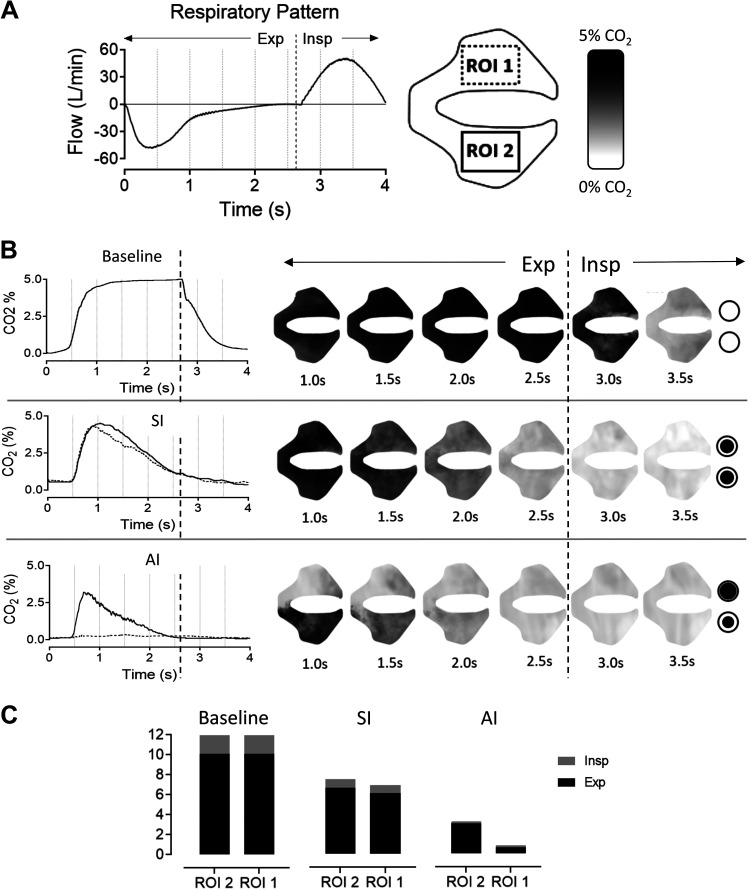
CO_2_ kinetics in an optical upper airway model during one breathing cycle, at an respiratory rate (RR) of 15 min^−1^ and nasal high flow (NHF) of 40 L/min. *A*: the breathing pattern used in the subsequent graphs. CO_2_ percentage was measured with infrared spectroscopy in rectangular regions of interests (ROI 1 and ROI 2). *B*: time graph along with the corresponding screenshots of both nasal cavities during a single breath. The vertical dotted line represents the time when expiration changes to inspiration. The dashed line on the graph represents the top cavity and the solid line the bottom cavity. The cavity with the larger asymmetrical prong had markedly less CO_2_ throughout respiration. *C*: using the area under the curve of the time graphs in *B*, the amount of CO_2_ in the nasal cavity during expiration (black) and inspiration (gray).

Figure 5 demonstrates the clearance of CO_2_ and gas kinetics in the nasal cavities during expiration with a RR of 15 min^−1^ and NHF at 40 L/min during normal breathing without NHF and with NHF via the SI and AI. An example of the normal respiratory pattern and the regions of interest used to calculate the CO_2_ concentration are portrayed in Fig. 5*A*. Infrared spectroscopy revealed the CO_2_ clearance in both nasal cavities with NHF. In Fig. 5*B*, CO_2_ clearance time graphs and snapshots from the optical model show CO_2_ clearance over time. Expired gas was purged with NHF during expiration. With the AI the nasal cavity with the large prong had low levels of CO_2_ throughout the breathing cycle and CO_2_ was purged through the less occluded nare (Supplemental Video S2). The SI produced more turbulent flow, forming eddies in both nasal cavities and purging CO_2_ via both nares. In Fig. 5*C*, the area under the curve is shown for both nares during expiration and inspiration. The difference in the amount of CO_2_ in the nasal cavity and the asymmetry between cavities with the AI can be seen clearly.

**Figure 6.**
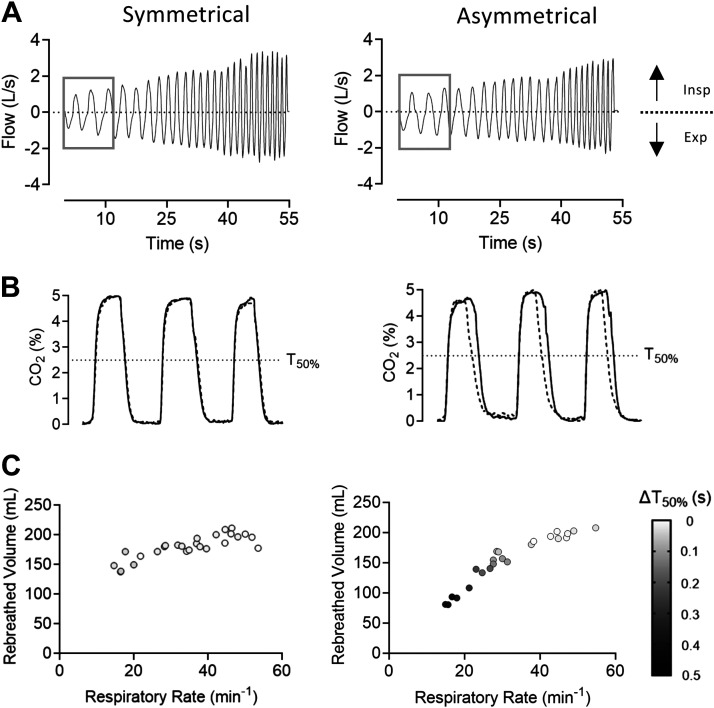
Breathing by an operator through the optical model via the symmetrical interface (SI) and the asymmetrical interface (AI) at nasal high flow (NHF) rate of 20 L/min. *A*: voluntary increase of respiratory rate (RR) over a minute. *B*: the first three breaths from *A* show the percentage of CO_2_ (measured by infrared spectroscopy) in each nasal cavity (solid line -> right nasal cavity, dotted line -> left nasal cavity). In the AI, the left prong has the larger diameter and the difference in *T*_50%_ can be seen by the left shift of the dotted line. *C*: multivariable plots demonstrate that a reduction in re-breathing (measured in the trachea) at a low RR with the AI was related to a difference in time to clear 50% of the CO_2_ (Δ*T*_50%_) in the respective nasal cavities.

A demonstration of an operator breathing through the optical model for 1 min with a steadily increasing RR is shown in Fig. 6*A*. CO_2_ concentration during slow breathing (RR ≈ 15 min^−1^) is depicted in Fig. 6*B* with the CO_2_ concentration in each nasal cavity presented. With the SI the CO_2_ concentration is approximately equal in both cavities. The CO_2_ concentration in the cavity with the large prong is lower in the AI as can be seen by the shift between the dashed and solid lines. Multivariable plots in Fig. 6*C* demonstrate the volume of dead space re-breathed for the SI and AI during increasing RRs. Reduced re-breathing measured at the trachea level corresponded to an increase in the difference in time it takes for CO_2_ to clear by 50% in each cavity (Δ*T*_50%_) measured by infrared spectroscopy during NHF with the AI.

### Differential Pressure between Nasal Cavities

**Figure 7.**
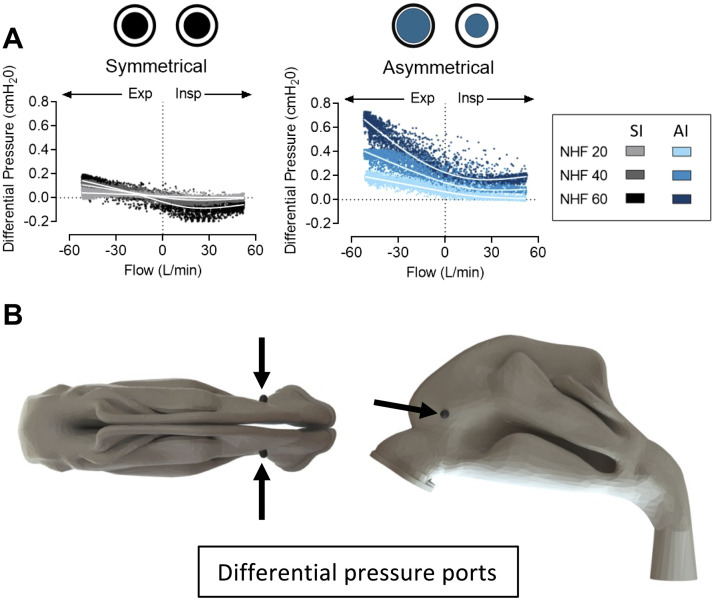
*A*: differential pressure measured in nasal cavities of the three-dimensional model during nasal high flow (NHF) rates of 20, 40, and 60 L/min via symmetrical interface (SI) and asymmetrical interface (AI) with a respiratory rate (RR) range from 15 to 45 min^−1^ across all breaths. LOWESS lines are fitted to each dataset. The pressure in cmH_2_O at flow rates of −0.1 and 0.1 L/min for the SI and the AI respectively are: 20 L/min 0.016 ± 0.005 vs. 0.066 ± 0.017, *P* < 0.001; 40 L/min 0.023 ± 0.011 vs. 0.120 ± 0.021, *P* < 0.001; and 60 L/min 0.007 ± 0.027 vs. 0.201 ± 0.019, *P* < 0.001. *B*: differential pressure ports in the model.

Differential pressure between nasal cavities measured in the three-dimensional model during NHF at 20, 40, and 60 L/min for all breaths measured at a RR between 15 and 45 min^−1^ is presented in Fig. 7. With the AI the pressure difference between nares was larger; it increased from inspiration to expiration and remained elevated between the breathing phases relative to the SI. The pressure difference around flow = 0 L/min at the end of expiration is higher for the AI when compared with the SI (NHF 60 L/min 0.201 ± 0.019 vs. 0.007 ± 0.027 cmH_2_O, *P* < 0.001).

### Mathematical Modeling

**Figure 8.**
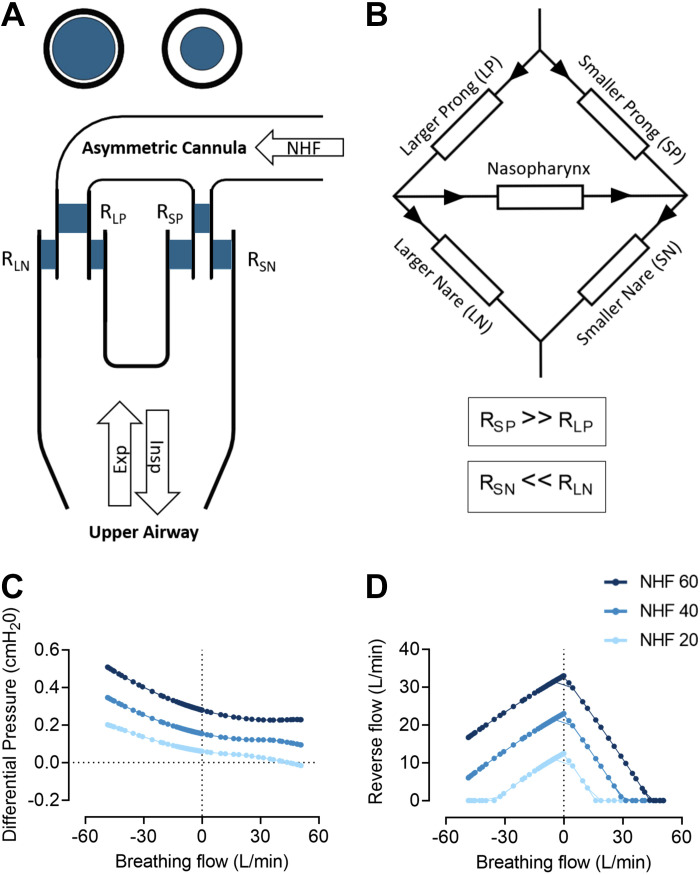
Mathematical modeling of flow in the upper airways with an asymmetrical interface (AI). *A*: a schematic view of upper airways with the AI demonstrates resistance in the larger prong (RLP), the nare occluded with the larger prong (RNL), smaller prong (RSP), the nare occluded with the smaller prong (RSN), nasal high flow (NHF), and tidal breathing flow (expiration and inspiration). *B*: the Wheatstone bridge circuit consisted of five resistors: resistance in the smaller prong was higher than in the larger prong and resistance in the nare occluded by the smaller prong was lower than resistance of the nare occluded by the larger prong, and the resistance in the nasopharynx was very small. During inspiration, the lowest resistance in the cannula was in the larger prong. During expiration, the lowest resistance was in the less occluded nare with the smaller prong, which created asymmetry and diverted the expired gas via the less occluded nare. *C*: NHF at 20, 40, and 60 L/min generated flow-dependent differential pressure between the nasal cavities during the breathing cycle. *D*: reverse flow between the nasal cavities peaked at the end of expiration and led to unidirectional purging of expired gas via the nare occluded by the smaller prong.

A mathematical model of flow and pressure in the nasal cavities during NHF with asymmetrically shaped prongs is presented in Fig. 8 and in the Supplemental Material. A schematic view of the model of the AI is presented in Fig. 8*A* and as a Wheatstone bridge circuit in Fig. 8*B*. During inspiration the lowest resistance was in the larger prong and during expiration, the lowest resistance was in the nare occluded with the smaller prong. This changed the flow direction during the breathing cycle and generated differential pressure between the nasal cavities, as shown in Fig. 8*C*. As a result, flow was generated across the nasopharynx via the choana. This reverse flow is presented in Fig. 8*D*.

**Figure 9.**
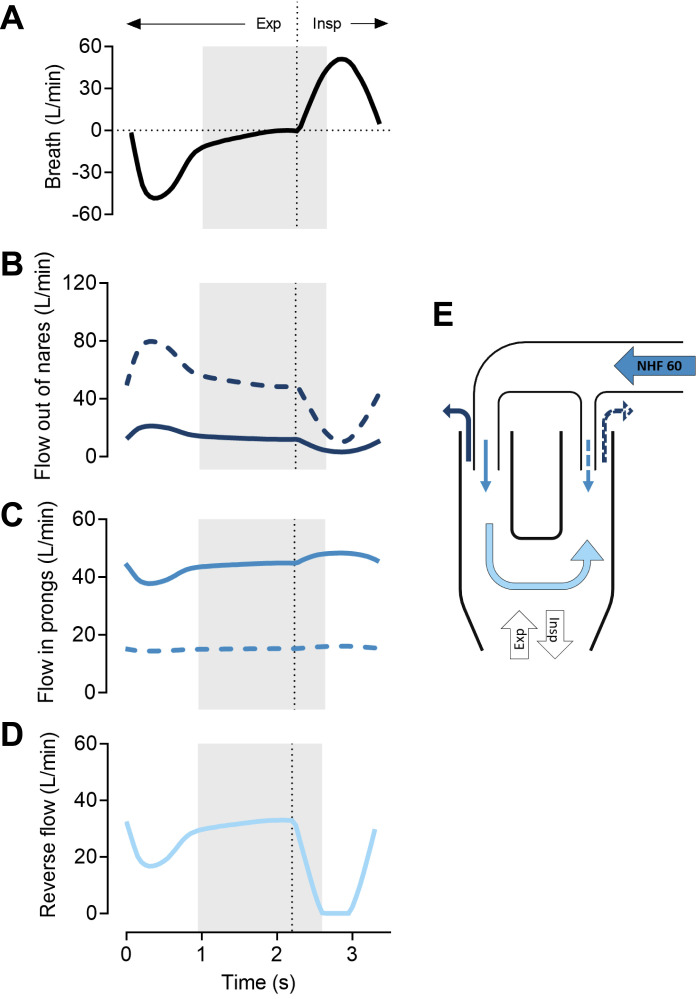
Flow within the nasal cavities and asymmetrical cannula prongs with nasal high flow (NHF) at 60 L/min during a single breath. The shaded area represents time for clearance as the last and the first 100 mL of expiration and inspiration, respectively. The vertical dotted line denotes the change from expiration to inspiration. *A*: flow of a single breath (respiratory rate 18 min^−1^, Ti:Te 1:2). *B*: flow out of the nares during a single breath (solid line = nare with the larger prong, dotted line = nare with the smaller prong). *C*: flow in the nasal prongs during a single breath (solid line = nare with the larger prong, dotted line = nare with the smaller prong). *D*: reverse flow through the choanae; this became maximal at the end of expiration and dropped during inspiration. *E*: schematic view of flows.

Mathematical modeling of flow within the nasal cavities and asymmetrical prongs with NHF at 60 L/min during a single breath is presented in Fig. 9. Figure 9*A* demonstrates the flow of breathing at an RR of 18 min^−1^ and Ti:Te 1:2. The graphs show flow within each nare (Fig. 9*B*) and the flow within the prongs (Fig. 9*C*). The difference in flow between the larger and smaller prong can easily be seen. The reverse flow between the nasal cavities was at its maximum at the end of expiration (Fig. 9*D*). Figure 9*E* demonstrates where the flows were measured and the reverse flow between nasal cavities.

## DISCUSSION

### Effect of Increased Asymmetrical versus Symmetrical Occlusion on Clearance of Dead Space

The results demonstrate that during NHF increasing the occlusion of the nares with an asymmetrically shaped nasal cannula (AI) led to improved clearance of dead space when compared with a similar occlusion with a symmetrically shaped cannula (SI). The AI performed significantly better when the breathing pattern had a reduced clearance time, due to elevated breathing frequency or with expiratory flow limitation. This is common in patients with acute respiratory failure and in patients with stable COPD (2, 7). Asymmetrical occlusion and flow produced by the different-sized prongs led to high and dynamic differential pressure and reverse flow across the nasal cavities, resulting in improved dead-space clearance. When one nare was more occluded the leak area was reduced and flow was biased to the contralateral side during expiration, which purged most of the expired gas via the less occluded nare with the greater level of leak. This unidirectional purging accelerated clearance of dead space in the upper airway and reduced re-breathing, even when there was a reduced time to clear.

The data from the anatomically correct three-dimensional upper airway model showed that enlarging the symmetrical nasal prongs led to lower clearance at high RRs. This confirms that a high RR increases re-breathing due to a reduction in clearance time (7). Higher total occlusion of the nares by the asymmetrically shaped prongs significantly increased clearance in the majority of respiratory patterns, with maximum differences in clearance achieved at lower NHF settings. Overall, with RRs of 35 and 45 min^−1^ the asymmetrical NHF led to an increase in dead-space clearance by 20% to 30% relative to NHF with the standard symmetrical cannula. This may substantially improve ventilation for patients with acute respiratory failure, where the ratio between dead space and tidal volume is elevated.

Increased total nare occlusion and lower overall leak around the prongs resulted in an elevated PEEP due to an increased level of resistance. A more linear relationship was seen between the clearance and PEEP with the SI at an RR of 45 min^−1^ than was seen with the AI at the same breathing frequency. Nonlinearity in the AI may be explained by a higher clearance efficiency at lower NHF rates.

With the AI at NHF 40 and 60 L/min, the difference in clearance between an RR of 15 and 45 min^−1^ was approximately half of the difference seen with the SI. This was observed both in the smaller and larger interface sizes where the larger prong almost completely occluded the nare. This suggests that the level of occlusion plays a lesser role in the clearance of dead space than the asymmetrical flow. Experiments with larger nares further confirm this, although the lower level of relative occlusion resulted in a lower PEEP, but the level of clearance was still improved (Supplemental Fig. S1).

At lower RRs, the breathing pattern can affect the dead-space clearance efficiency of NHF therapy. Patients with COPD may have a substantially lower clearance even when the expiratory time is increased (21, 22). This is caused by a reduction in time that corresponds to the last and first 100 mL of expiration and inspiration respectively, where most of the dead-space clearance may occur (7). The simulated COPD breathing pattern with an intrinsic PEEP (COPD 2) had the same Ti:Te ratio (1:2) as the control but it had significantly reduced the level of clearance. This can be explained by a rapid change between expiratory and inspiratory flow. However, the increased asymmetrical occlusion significantly improved the amount of clearance even at NHF of 20 L/min.

When compared with the SI with a similar area of occlusion, the AI improved clearance in both the control and COPD groups, as it did with an increasing RR. At a low RR and a NHF rate of 40 or 60 L/min, clearance was similar between interfaces, unless time for clearance was shortened as was the case with expiratory flow limitation. There was no significant difference in clearance when comparing the AI at NHF 20 L/min and the SI at 60 L/min at all breathing frequencies investigated. The AI increased clearance threefold at NHF 20 L/min compared with the SI in the COPD group with expiratory flow limitation and intrinsic PEEP. So, when using the AI a similar degree of clearance can be achieved with a much lower NHF rate. High efficiency of dead-space clearance may play an important role in the treatment of chronic patients receiving NHF therapy, as low flow is generally better tolerated (23).

### Kinetics of Dead-Space Clearance during Asymmetrical Occlusion

The experiments with an optically transparent upper airway model demonstrated that the accelerated clearance of CO_2_ was related to asymmetry in the kinetics of clearance. The cavity that was occluded with the larger prong remained mostly free of CO_2_. NHF reduced the end-tidal CO_2_ with symmetrical occlusion, which started to be purged in both cavities before the end of expiration. With the AI, purging of CO_2_ started earlier in the breathing cycle. This was further confirmed by an experiment where an operator breathed through the model to demonstrate the effect of variable respiration. This showed that the re-breathing volume per breath was directly related to breathing frequency. With the AI the cavity with the large prong began clearing earlier in the breathing cycle, which led to the difference between the time of cavity clearance (Δ*T*_50_ [CO_2_]). Slow-motion playback (×0.050) of video recordings of CO_2_ kinetics during NHF with the asymmetrical occlusion showed purging of expired CO_2_ via the less occluded nare (Supplemental Video S2).

### Mathematical Modeling

Resistance to flow in the nasopharynx is relatively small due to the large cross-section of the choanae. Symmetrical flow resistance, both inside the prongs and in the nares, results in no flow across the nasal cavities. The AI causes higher resistance to flow both inside the smaller prong and in the more occluded nare that is occluded by a larger prong. Resistance is lower inside the large prong and in the less occluded nare by the smaller prong. NHF becomes asymmetrical due to the different diameters of the prongs causing the Wheatstone bridge to become unbalanced, which leads to pressure differences between the nasal cavities. This was confirmed by the three-dimensional bench-top model. The differential pressure generates reverse flow in the nasopharynx from the more occluded to the less occluded nare to balance the bridge circuit, which resembles the pendeluft effect observed in the bronchial tree (24, 25).

**Figure 10.**
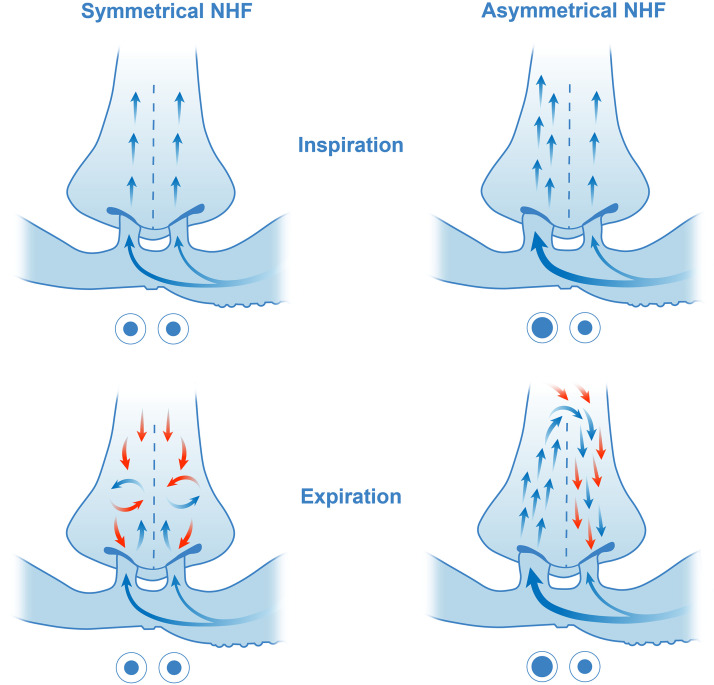
Schematic representation of the flow direction in cannulae and the upper airways during inspiration (*top*) and expiration (*bottom*) in a symmetrical interface (SI) (*left*) and an asymmetrical interface (AI) (*right*). Blue arrows indicate nasal high flow (NHF), which is equally split between the prongs in the SI. In the AI, NHF is biased toward the larger prong due to its lower resistance and the streamline of gas velocity within the cannula. Expired gas flow is indicated by red arrows. During expiration, the SI leads to equal mixing and purging via both nares. In the AI, the nare occluded by the smaller prong creates a lower resistance path for the expired gas to be cleared from the nasal cavity. The biased flow from the larger prong is also directed to the contralateral nasal cavity via the choanae, forming the reverse flow that peaks at the end of expiration.

The flow distribution between the prongs varied over the breathing cycle. During inspiration, flow was higher through the larger prong; during expiration, flow was higher through the nare occluded with the smaller prong. The flow direction within cannulae and the upper airways during inspiration and expiration with the SI and AI is schematically demonstrated in Fig. 10. With larger nares the resistance to flow during expiration will not be substantially different; therefore, the variation in flow distribution from the prongs will be smaller. However, the AI will always lead to asymmetrical flow and reversed flow between the nasal cavities, with the magnitude directly related to the cross-sectional area of the prongs in relation to the area of the nares.

### Limitations

The three-dimensional model did not fully represent the shape of the nares in the human airways. The lack of soft palate, vocal cords, and other structures could lead to an overestimation of dead-space clearance compared with what may occur in situ. Calibration to baseline was performed for every RR and breathing pattern; consequently, differences in clearance between breathing frequencies, presented as normalized data, could be exaggerated. However, findings shown previously are similar, where a rise in the RR increased re-breathing (7). In addition, when CO_2_ was sampled directly from the trachea in tracheostomized patients on NHF at 45 L/min, a reduction of 3 mL of CO_2_ per breath was found, reducing dead space by ∼60 mL (14).

The open mouth may influence dead-space clearance and positive airway pressure during NHF (26, 27). The effects of the open mouth on ventilation could depend on the biomechanics of the soft palate, which is responsible for the partitioning of oronasal flow (28). A position of the soft palate is regulated by a complex interaction between six muscles (the velopharyngeal constrictor, the levator veli palatini, the superior pharyngeal constrictor, the velopharyngeal dilator muscles, the palatopharyngeus, and the palatoglosus) and can be affected by resistive loading, pressure oscillations, and hypoxic hypercapnia (29–31). In a physiological study on newborns, an opening of the mouth by an investigator during NHF and CPAP did not affect pressure in the nasal cavities and minute ventilation (10). The findings cannot be directly extrapolated to the adult population, but they demonstrate that the open mouth does not necessarily create a leak that could lead to a reduction in pressure, or alter rebreathing of expired gas. The aforementioned indicates that interactions between nasal and oral breathing during NHF or other forms of noninvasive respiratory support need to be studied in vivo.

Results may further be influenced by the variability in nare cross-sectional area, as this affects occlusion and varies substantially within the human population. The averaged minimal cross-sectional area that was used in the model has been recently confirmed in a study using computer tomography in an adult European population (18). There is a considerable anatomical variability of the upper airway related to ethnicity and gender. Caucasians typically have a narrow nose (leptorrhine), which is very different from flat (platyrrhine) African and intermediate (mesorrhine) Asian noses (32). A recent study investigated the impact of morphological variations of the nose on the nasal function and found that median resistance was highest among Caucasians and lowest among East Asians. Overall, East Asian and black females had higher nasal resistance when compared with males of the same ethnicity, whereas in Latinos and Caucasians the males have higher resistance (33). However, the absolute values of nasal resistance are irrelevant here as they will affect the magnitude but not the mechanisms of dead-space clearance with asymmetrical flow.

Nasal cycling is another source of variability that may affect resistance in the nares. The phenomenon is characterized by periodically alternating resistance between nasal cavities caused by the obstruction of the nasal passages by erectile tissue (34). Most people exhibit a nasal cycle dynamically changing resistance every few hours, however the extent and pattern can vary and is often exaggerated during upper airway pathology (35). The asymmetrical occlusion depends on the cross-sectional area of the prongs relative to the vestibulum nasi, which is the area just inside the nostril at the entrance of the nasal cavity. The nasal cannula does not extend to the internal nasal valve area beyond the vestibulum nasi. Changes in resistance caused by the nasal cycle may affect clearance of dead space and the pressure generated during NHF. The nasal cycle as well as other factors that may influence nasal flow and resistance, such as nasal valve stenosis, collapse, septal deviation, turbine hypertrophy, or sino-nasal inflammatory disorders, needs to be addressed in further physiological studies focusing on nasal patency (36).

Nasal decongestants may affect NHF therapy by changing nasal resistance, but this was not addressed in this current investigation. A study using high-resolution magnetic resonance imaging and computational flow dynamics in healthy subjects found that decongestants increased the averaged cross-sectional area of the nasal cavity by a factor of 1.4 and halved the nasal resistance (37). This may again affect the magnitude but not the mechanism of clearance with asymmetrical flow.

None of the limitations raised in this section have been mentioned as concerns in the literature. Only a bench-top study by Marshall et al. (38) showed a significant decrease in delivered O_2_ when nasal patency went from bilateral to unilateral with a low-flow cannula at 0.5–6.0 L/min, but this is unlikely to affect clinical outcomes across the full range of flows.

The current study did not investigate interfaces with a single prong during NHF. However, the models used demonstrated that NHF with a single prong should also improve clearance by producing reverse flow between the nasal cavities, although less pressure is generated due to a reduction in contralateral occlusion. The use of smaller nasal cannulae was not included in this investigation as the occlusion of the nares would be reduced and, therefore, it is unlikely to lead to increased airway pressure. Furthermore, a recent study suggested that small-bore nasal cannulae generate a jetting effect and can result in undue shear stress on the mucosa, leading to epistaxis in 7 of 70 patients (39). It should be noted that NHF rates in this study did not exceed 40 L/min.

The optical model used is oversimplified, and it does not fully represent the complexities of the nasal cavities, but it was a necessary compromise for investigating CO_2_ kinetics during NHF and helped to visualize the reverse flow derived from the mathematical modeling. Further analysis showed that there was a strong correlation in the improvement in the percentage dead-space cleared between the optical and anatomical models (Supplemental Fig. S2). This means that while the models used had limitations, the mechanisms remain valid, and the anatomical complexities may not bear greatly on the clearance mechanism.

### Clinical Significance

These experiments may not be directly extrapolated to the clinical settings, but they demonstrate a substantial difference in dead-space clearance between the SI and AI when time for clearance is reduced.

Positive airway pressure during noninvasive respiratory support plays a substantial role in managing patients with acute respiratory failure. Morais et al. (40) demonstrated that the effort-dependent lung injury in patients with severe ARDS can be minimized by an increased PEEP. A recent multicenter randomized clinical trial in patients with hypoxemic respiratory failure, due to COVID-19, showed that CPAP significantly reduced the risk of tracheal intubation compared with conventional O_2_ therapy. NHF did not differ from conventional oxygenation in this study, but the authors admitted to being underpowered for such a comparison (41). Nevertheless, lower pressure support with NHF versus CPAP in critically ill patients with COVID-19 may have an impact on the outcome. Positive airway pressure was also addressed in a study where NHF was combined with a helmet interface that maintained CPAP while on NHF (42). In this physiological study, the authors controlled PEEP at 3, 5, and 8 cmH_2_O with the helmet during NHF 50 L/min in healthy volunteers. These pressures were able to be maintained, but the complex setup made clinical application questionable. NHF with the AI may be able to provide a clinically significant level of PEEP without a sealed interface such as a mask or helmet.

Positive airway pressure may be increased by larger symmetrical prongs, but this can potentially lead to a complete seal of the nares and generate uncontrolled pressure (7). With the use of an AI, the risk of a total seal and uncontrolled pressure can be mitigated. Even if the larger prong produces a complete seal, the smaller prong will allow for leak. Therefore, increased occlusion with the AI can increase airway pressure, as well as dead-space clearance through asymmetrical flow. This reduces re-breathing and is associated with an improvement in the work of breathing (2, 7, 27). Previously it was speculated that the larger cannula interface is likely to affect the clearance of dead space as the nasal cavity volume is small relative to NHF (9). A more recent study demonstrated that clearance of dead space is in fact inversely correlated with RR due to the reduced time for dilution and purging of expired gas with greater occlusion (7). The present investigation expands on this, demonstrating that aside from an increased RR the breathing pattern may also reduce efficiency of clearance, particularly when expiratory flow at the end of the expiratory phase is high and quickly changes direction with the start of inspiration. This is relevant to patients with expiratory flow limitations, and especially with intrinsic PEEP, which leads to hyperinflation. These patients have less time available for dead-space clearance, even at low RRs. In patients with hypercapnic COPD, who often present with expiratory flow limitation and intrinsic PEEP, a reduction in re-breathing is associated with a decrease in tissue and arterial CO_2_ (43, 44).

A reduction of re-breathing with unidirectional flow was also described by Jiang et al. (45), who found that the unidirectional flow during pursed-lip breathing attributes to a significant reduction in functional anatomical dead space and improvement in breathing efficiency. Later it was suggested that the improved breathing efficiency could be associated with benefits for patients with COPD (46).

In agreement with previous work, at a low RR, clearance is efficient with lower NHF rates (7). The current investigation also found that with an RR of 15 min^−1^ during normal breathing (Ti:Te 1:2), the difference between the SI and AI was significant at low NHF, as both were efficient at high NHF. Patients in critical care with acute respiratory failure and a high RR that require noninvasive respiratory support may benefit from NHF with increased positive airway pressure. The use of the AI showed significant improvement in dead-space clearance at high RRs. With simulated expiratory flow limitation and low RR, clearance with NHF was reduced due to a fast transition from expiration to inspiration; the AI significantly improved clearance in this scenario even at a low NHF setting. Patients with expiratory flow limitations, like those with stable COPD, are usually on low NHF to improve tolerance; with the AI, NHF could be set to a lower flow while maintaining clearance (47).

### Conclusions

During NHF, increased nare occlusion using an asymmetrically shaped cannula interface elevated the airway pressure and improved dead-space clearance. This improvement in clearance was highest in breathing patterns with increased breathing frequency or with expiratory flow limitation, where less time for clearance was available. Asymmetrical NHF generates reverse flow between the nasal cavities, which accelerates the purging of expired gas via the less occluded nare. The findings need to be assessed in clinical settings.

## DATA AVAILABILITY

Data will be made available upon reasonable request.

## SUPPLEMENTAL DATA

10.6084/m9.figshare.21520728 Supplemental Video S1: https://doi.org/10.6084/m9.figshare.21520728;

10.6084/m9.figshare.21520827 Supplemental Video S2: https://doi.org/10.6084/m9.figshare.21520827;

10.6084/m9.figshare.21514605 Supplemental Fig. S1: https://doi.org/10.6084/m9.figshare.21514605;

10.6084/m9.figshare.21520578 Supplemental Fig. S2: https://doi.org/10.6084/m9.figshare.21520578;

10.6084/m9.figshare.21520689 Supplemental Material: https://doi.org/10.6084/m9.figshare.21520689.

## GRANTS

The work was fully supported by Fisher & Paykel Healthcare Ltd.

## DISCLOSURES

S.T., M.R., A.G., and L.G.T.v.d.H. are employees of Fisher & Paykel Healthcare. S.T. disclosures a US Patent No. 10569043: Asymmetrical nasal delivery elements and fittings for nasal interfaces.

## AUTHOR CONTRIBUTIONS

S.T. and G.N. conceived and designed research; M.R. and A.G. performed experiments; M.R., A.G., and L.G.T.v.d.H. analyzed data; S.T., L.G.T.v.d.H., and G.N. interpreted results of experiments; M.R. prepared figures; S.T. drafted manuscript; S.T., M.R., A.G., L.G.T.v.d.H. and G.N. edited and revised manuscript; S.T., M.R., A.G., L.G.T.v.d.H., and G.N. approved final version of manuscript.
